# Advancing the understanding of allergic contact dermatitis: from pathophysiology to novel therapeutic approaches

**DOI:** 10.3389/fmed.2023.1184289

**Published:** 2023-05-22

**Authors:** Marta Tramontana, Katharina Hansel, Leonardo Bianchi, Chiara Sensini, Nicolò Malatesta, Luca Stingeni

**Affiliations:** Dermatology Section, Department of Medicine and Surgery, University of Perugia, Perugia, Italy

**Keywords:** allergic contact dermatitis, allergens, patch test, delayed-type hypersensitivity, eczema, inflammatory skin diseases

## Abstract

Allergic contact dermatitis (ACD) is a common inflammatory skin disease that, especially when the condition becomes chronic, has a high impact on the quality of life and represents a significant disease burden. ACD represents a type IV delayed-type hypersensitivity reaction that is triggered by contact with an allergen in previously sensitized individuals through the activation of allergen-specific T cells. In the acute phase, it is characterized by eczematous dermatitis, which presents with erythema, edema, vesicles, scaling, and intense itch. Non-eczematous clinical forms are also described (lichenoid, bullous, and lymphomatosis). Lichenification is the most common clinical picture in the chronic phase if the culprit allergen is not found or eliminated. ACD can be associated with both occupational and non-occupational exposure to allergens, representing approximately 90% of occupational skin disorders along with irritant contact dermatitis. Patch testing with suspected allergens is required for a diagnosis. Metals, especially nickel, fragrance mix, isothiazolinones, and para-phenylenediamine, are the most commonly positive allergens in patients patch tested for suspected ACD. The treatment goal is to avoid contact with the culprit agent and use topical and/or systemic corticosteroid therapy.

## 1. Introduction

Contact dermatitis (CD) refers to an inflammatory skin condition that occurs after exposure to an exogenous substance (1, 2). Irritant contact dermatitis (ICD) and allergic contact dermatitis (ACD) are the two subgroups of CD which differ in both clinical an pathophysiological aspects (3). Additional CD forms comprise protein CD and photo contact dermatitis, including photoallergic and phototoxic CD (4). Mucous membranes can also be involved. ICD is the most frequent form of CD (approximately 80% of cases of CD are ICD), and it is due to inflammatory and cytotoxic effects induced by exposure to several environmental agents (physical or chemical) that can activate the innate immune system (2, 4). The clinical pictures of ICD are related to different factors, such as the concentration and chemical properties of the irritant agent, the duration and frequency of the contact, environmental conditions, and skin features. The latter is related to the patient’s age, gender, and cutaneous susceptibility (for example, the presence of other skin conditions) (4, 5). Innate immune system activation, skin barrier changes, and T lymphocyte recruitment are implicated in the pathomechanism of ICD (4). Water, soaps, solvents, and detergents are common irritant agents, while hands, especially finger web spaces, are the most frequent sites involved. Xerosis and scaling represent the most common clinical features of ICD. The diagnosis is often based on a detailed clinical examination and patient history.

Allergic contact dermatitis approximately comprises 20% of cases of CD (3). It is a type IV delayed hypersensitivity reaction triggered by skin contact with an allergen only in previously sensitized subjects. Reexposure to the same substance elicits an immunologic reaction by migrating circulating memory T cells into the skin, causing cutaneous inflammation within 48 h (2, 3). The most common clinical feature of acute ACD is eczematous dermatitis, characterized by erythema, edema, papules, vesicles or bullae, oozing, and crusts. Typically, lesions are ill defined, spreading beyond the contact site of the allergen, while ICD is usually characterized by well-defined margins (1, 3, 6). Itching is one of the main symptoms of ACD and is always referred by patients. Non-eczematous forms of ACD are also reported, such as lichenoid, purpuric, erythema-multiforme-like, bullous, pigmented, lymphomatoid, granulomatous, or pustular ACD (1, 6). Although ACD can be localized anywhere on the body, the hands are the most common site (1). Nickel, fragrance mix, methylchloroisothiazolinone/methylisothiazolinone, and para-phenylenediamine are considered the most common allergens inducing ACD (1–4). Patch testing is the gold standard for diagnosing ACD, and topical and systemic corticosteroids represent the first line of treatment for this condition.

Allergic contact dermatitis and ICD can be related to occupational or non-occupational exposure to allergens or irritants, and the avoidance of contact with the culprit agent is the goal of treatment. If the causative substance is not found or removed, dermatitis tends to become chronic and cause significant patient disability, negatively impacting the quality of life.

The present manuscript summarizes the epidemiology, pathomechanism, clinical features, culprit allergens, diagnosis, and treatment of ACD.

## 2. Epidemiology

It is estimated that between 4 and 7% of all dermatology consultations annually are for CD (1). It can occur at any age and in both genders, although it is more common in women, especially if employed in household activities. The exact prevalence of CD is unknown, but it is estimated to range between 1.7 and 9.8%, according to several published studies (1, 7, 8). Recent literature data point out a prevalence of contact allergy between 15.0 and 20.1% of patch-tested patients in the general population (9–11). Occupation is the main risk factor for CD, and 90% of occupational skin disorders in the industrialized world are CD, particularly ICD (1, 8). It is estimated that hairdressers, wetworkers, food handlers, health care workers, and building and metal workers are more likely to develop contact allergies due to repeated exposure to the most common allergens (12).

Allergic contact dermatitis can cause a significant disability, contributing to lengthy leaves of absence and negatively impacting workplace productivity and the socioeconomic state of the patient and the entire society (12). The literature about an increased prevalence of ACD in atopic dermatitis (AD) patients is quite discordant, with some studies reporting an increased prevalence of contact sensitization in AD subjects and others documenting reverse data (13, 14). A recent systematic review of the association between AD and contact sensitization showed that AD patients have a frequency of contact allergy similar to the general population, recommending that patch testing in AD individuals should be considered only when ACD is suspected (15).

## 3. Pathomechanism

Allergic contact dermatitis is a type IV delayed-type reaction that needs an initial sensitization phase with a previously innocuous substance (16). Two phases can be distinguished: an afferent and an efferent phase. The first phase is sensitization, which occurs when a foreign substance penetrates the skin and binds to skin proteins, inducing the formation of an antigen complex (3, 10, 12). This process stimulates an inflammatory reaction with innate immunity activation through keratinocyte release of several cytokines, such as IL-1α, IL-1β, tumor necrosis factor-α, IL-8, IL-18, granulocyte-macrophage colony-stimulating factor (12). Allergens are then uptaken by antigen-presenting cells (APCs), such as Langerhans cells and dermal dendritic cells, and migrate to the regional lymph nodes, where antigen-specific T cells (Th1, Th2, Th17, and T regulatory cells) are activated and subsequently proliferate and circulate in the blood. The naïve T cells, recognizing specifically the complex allergen-major histocompatibility complex molecule, are activated, inducing the formation of effector and memory T cells.

Upon reexposure to the allergen, in the elicitation phase, the hapten-specific T cells recognize the allergen, activating an inflammatory reaction mediated by cytokines such as interferon γ (IFN-γ), IL-2, and IL-17 and producing a cellular infiltrate responsible for a clinical feature of ACD (12, 17).

The most recent studies showed that the immune response in ACD is hapten-specific, with both Th1 and Th2 responses. Nickel, for example, activates predominately Th1 and Th17-mediated pathways, while rubber and fragrances induce mainly Th2-mediated pathways (17).

## 4. Clinical features

Clinical signs of ACD can be characterized by polymorphic skin features. Differences may depend on the chemical characteristics of the culprit agent, the type and way of exposition, and the clinical and anatomical features of the affected skin area. The main symptom is itching, which may occur even within the first 24 h after exposure to an allergen. Although burning and pain are more typical of ICD (1, 3–6), they may also occur in rare cases of ACD. The most common clinical presentation of ACD is eczema, which requires about 5–7 days after first contact with the culprit allergen, while subsequent contact needs about 24–48 h to elicit a cutaneous reaction (6, 18).

During the acute phase, erythema, edema, and vesiculation (sometimes bullae, depending on the severity of the allergic reaction) can be observed. Erythema is characterized by a bright red or pinky-red color and ill-defined borders. Oedema is usually more severe when the face, eyelids, and genitalia are involved because of the high laxity of cutaneous and subcutaneous tissues at these sites. Vesicles, which usually develop a few hours after erythema and edema, tend to break quickly due to their superficial localization in the epidermis, resulting in multiple confluent erosions with, in severe cases, abundant oozing. In the subacute phase, erythema decreases, and small friable detachable crusts and pityriasis scales occur. If the culprit allergen persists because it has not been found or eliminated, a chronic phase may occur with the development of lichenification, characterized by the accentuation of skin grooves (1, 3, 6, 18).

Eczema develops in the area of the body where contact with allergens occurs but often extends over the application site with poorly defined margins. Lachapelle et al. (6) described two further stages of the progression of ACD. In the second stage of ACD, local dissemination of signs and symptoms may appear via lymphatic vessels with the development of erythematous or erythematous-vesicular, rarely erythema multiforme-like, lesions, which are usually less pronounced than those located at the primary site. In the third stage, hematogenous dissemination of antigen may lead to two different clinical features: the 3A stage, called “idic reaction,” and the 3B stage, or “systemic contact dermatitis.” Idic reactions induced by contact with an allergen, named “chemides” by Malten (6), are symmetric erythemato-oedematous, rarely desquamative or vesicular lesions, with pompholyx-like aspects on palms and soles. Systemic contact dermatitis is rather characterized by a symmetric cutaneous eruption that occurs after the systemic assumption of an allergen to which the subject was formerly sensitized through the skin (6).

The clinical presentation of ACD may sometimes be particularly challenging, mimicking ICD or other dermatological diseases. For this reason, a detailed medical history, an accurate physical examination, and allergy diagnostic tests are always required. Table 1 summarizes the main criteria for differential diagnosis between ACD and ICD.

**Table 1:** Differential diagnosis between allergic contact dermatitis (ACD) and irritant contact dermatitis (ICD).

Criteria	ACD	ICD
Occurrence	Skin lesions develop usually 5–7 days after first contact with allergen or 24–48 h after a further contact	Skin lesions appear few hours after first contact with substance
Symptoms	Itch, usually strong	Burning, rarely itch
Limits of lesions	Blurred. They tend to overcome the area of contact with allergen	Well defined, they often trace the shape of the irritant product and are limited to the contact area
Morphology	Acute eczema (erythema, oedema, vescicles) or dry eczema (erythema and desquamation)	Erythema and desquamation
Idic eruptions	May be present	None
Skin test	Positive patch test for culprit allergen	None
Histopathology	Spongiform dermatitis	Necrosis, acantolysis, intraepidermical bullae due to caustic damage to epidermis

Airborne CD and photoallergic CD represent two other types of ACD. Airborne CD may be caused by several volatile allergens. Clinically, it presents as classic contact eczema, which develops in areas that are exposed to the air. The most commonly involved sites are the face, neck, neckline, hands, wrists, arms, and eyelids (that may also be oedematous), behind the ears, and under the jaw regions. Skin lesions may be accompanied by tearing, photophobia, and conjunctival erythema. If an allergen is made of solid particles able to slip into clothes, eczema may also involve body areas that are covered by clothes, and it tends to develop into flexural folds. Plants are the most common culprits in airborne CD, especially those in the Asteraceae family. Other common agents are quinones (Tectona, rosewood), phenols (Anarcardiaceae), and terpenes (Frullania, Pinus). Clinical manifestations usually resolve a few days after the elimination of exposure to the allergen (18–20).

The photoallergic CD is a type IV delayed-type cutaneous reaction induced by skin contact with a photo antigen in previously sensitized subjects and triggered by ultraviolet radiation. It affects cutaneous areas exposed to the sun and, conversely, for airborne contact dermatitis, sites such as behind the ears and under the jaw regions are usually spared (18). Skin lesions that commonly appear within 24 h after sun exposure usually appears as a very itchy eczematous dermatitis with possible spreading eruptions. A diagnosis can be performed using a photo patch test, which will be discussed later. Chronic actinic dermatitis (CAD), also known as actinic reticuloid (AR), is a possible complication of photoallergic contact dermatitis, characterized by abnormal persistent photosensitivity despite the removal of the causative agent. The most common photo antigens are topical products, such as fragrances, antimicrobials, and nonsteroidal anti-inflammatory drugs (NSAIDs) (18, 20).

Finally, non-eczematous patterns of CD are also described, both for ICD and ACD.

### 4.1. Nummular ACD

Erythemato-papular-vesicular lesions characterized by coin-shaped and well-defined margins, typical of nummular eczema, may be an atypical presentation of contact allergy. They are usually localized on the arms and the dorsal hands, with sizes ranging between 1 and 5 cm. Rarely, it may be induced by nickel, and nummular AD is a differential diagnosis (18).

### 4.2. Bullous ACD

This form is characterized by blisters localized at the contact site with the culprit allergen. Histopathologic examination with direct immunofluorescence may be performed to exclude bullous pemphigoid or other skin autoimmune bullous disorders. This non-eczematous form is often caused by sensitization with cinnamic aldehyde, cinnamyl alcohol, thimerosal, and bufexamac and is not infrequently associated with a positive patch test with the culprit agent characterized by a vesicular or bullous reaction (1).

### 4.3. Pigmented ACD

It presents with marked spotted or reticular pigmentation during the acute phase of eczema, which is usually long-lasting even after the resolution of dermatitis. It is more frequent in Japan, where it is considered a clinical entity of its own (20). Azo-dyes, gums, and cinnamic alcohol derivatives are the allergens most commonly associated with this form. Some dermatologic conditions, such as Rielh’s melanosis and poikiloderma of Civatte, are considered forms of pigmented ACD, both of which are associated with sensitizing fragrances (1). In this variety of ACD, a positive patch test may induce a hyperpigmented area after the disappearance of an eczematous reaction.

### 4.4. Purpuric ACD

It is characterized by petechial and purpuric lesions, usually involving the lower limbs, especially the ankles. Histological examination shows extravasation of erythrocytes inside keratinocytes. Isopropyl-N-phenyl-p-phenylenediamine is considered the most common culprit allergen. It can also be associated with sensitization to balsam of Perù, topical NSAIDs, and kinase inhibitors (1, 20).

### 4.5. Lichenoid ACD

This is a lichen planus-like or lichenoid adverse drug reaction-like type of ACD. A diagnosis is usually performed after histological examination, which shows epidermal spongiosis and eosinophilia associated with dermal lichenoid band infiltration with mild inflammatory infiltration. A typical presentation of this variety is allergic contact stomatitis due to mercury orthodontic amalgam, often presenting as an erosive lichen planus of the peri-implant mucosa. Multiple allergens may be involved in this kind of ACD, such as *p*-phenylenediamine, chromates, cobalt, nickel, and palladium (20).

### 4.6. Linfomatoid ACD

It is characterized by clinical and histopathological features resembling mycosis fungoides, with papula-nodular lesions at the contact site associated with an intense itch that improves after avoiding the culprit allergen. Patch testing and spongiotic dermatitis, with the absence of atypical lymphocytes at a histopathological examination, allow a differential diagnosis. Gold, nickel, cobalt, and kaolin are typically associated with this subtype of ACD (1, 20).

### 4.7. Pustular ACD

Usually associated with textile dyes such as disperse blue and disperse red, pustular ACD presents an erythematous area with non-follicular sterile pustules localized at the contact site with an allergen. A patch test usually shows a pustular reaction, rarely a vesicular one (1).

### 4.8. Granulomatous ACD

It clinically presents as shiny papular or nodular lesions, ranging from reddish to brown, which develop 4–6 weeks after exposure to the culprit allergen following an initial vesicular eruption. Histopathological examination shows sarcoid granulomas, making it almost impossible to differentially diagnose sarcoidosis. Zirconium-containing deodorants are associated with this variety of ACD. Other causative agents are chrome, cobalt, cadmium-containing tattoo dyes, aluminum hydroxide-containing vaccines, mercury, beryllium, gold, palladium, and titanium-containing pacemakers (1).

## 5. Allergens

Nickel sulfate is still considered the most common allergen, followed by methylisothiazolinone, fragrance mix, formaldehyde, and p-phenylenediamine.

### 5.1. Nickel sulfate

Nickel sulfate is malleable, ductile, and resistant to atmospheric agents. Metal is used to cover other metals for protective or decorative purposes. In addition to other metals (iron, copper, chromium, and zinc), it forms alloys and is also used in coin manufacturing. It is one of the most widely used and marketed metals in the world. the allergen with the highest prevalence of contact allergy and the most commonly positive hapten in patients patch tested for suspected ACD. The risk of developing a nickel allergy is very high because nickel is a ubiquitous metal used in manufacturing several daily-use objects. Moreover, the risk seems to be facilitated and increased by the fast oxidation of this metal after contact with exudates of the skin or mucous membranes (21). Retrospective data collected in patch-tested patients with European baseline series in 2013/2014 by the European Surveillance System on Contact Allergies (ESSCA) showed that nickel was the allergen with the highest frequency of positive reactions (18.1%) (22). Especially, Italy was the state with the highest prevalence of nickel ACD among European countries. In 2008, nickel was designated as the contact allergen of the year by ACDS (23).

Nickel sensitivity occurs more frequently among women, with an F/M ratio ranging from 3:1 to 14:1.9 because of the higher number of exposure sources to the metal in women than in men, with an estimated prevalence, respectively, of 15.7% in females and ranging from 2 to 8% in males (24). In the latter, sensitization seems to occur mainly in occupational settings (9, 25).

Jewellery represents the most common exposure source for nickel allergy and includes earrings, necklaces, medals, brooches, bracelets, watches, rings, anklets, jewelry used for ear piercing, and other body parts. Jewelry: earrings (commonly called “hypoallergenic”), necklaces, medals, pins, bracelets, watches, rings, anklets, jewelry used for ear piercing, and other body parts.

Gold, especially white gold, may also contain nickel, while yellow gold generally contains a very low amount. Moreover, silver is frequently used in alloys with nickel in jewelry items, so its use is not recommended. Metal accessories for clothing, such as jeans buttons, zippers, anklets, necklaces, armlets, watch straps, hair clips and pins, hair curlers, metal parts of glasses, and watch cases, represent another important source of exposition (24).

Cosmetics, mainly pigmented mascara, eyeshadow, soaps, and detergents, may contain nickel traces, industrial cutting liquids, and kitchen utensils. Finally, medical devices (orthopedic joint replacement, heart and endovascular prostheses, surgical instruments, and metal dental braces) can be considered as possible occupational and non-occupational exposure sources of nickel (23, 24).

### 5.2. Methylchloroisothiazolinone/methylisothiazolinone and methylisothiazolinone

The mixture of methylchloroisothiazolinone/methylisothiazolinone (MCI/MI; trade names: Kathon CG, Euxyl K 440) in the form of a 3:1 mixture is one of the most widely used preservatives in industrial, cosmetic, and household products (26).

In the group of cosmetics and personal care products, MCI/MI can be found in products such as detergents, shampoo, conditioners, cleansing wipes, make-up remover, facial and body creams, deodorants, foundation, mascara, eye shadow, dyes, fixers, shaving products, and sunscreen.

Among industrial-use products, it can be contained in cutting oils and lubricants in the metalworking industry, wall paints, water-based dyes, adhesives, paints, polishes, toners, printing inks, and industrial detergents.

In addition, several detergents and softeners for linen and housecleaning products can include MCI/MI in their composition (26–28).

The rates of allergic contact dermatitis to MCI/MI have increased significantly in the last 40 years, concurrently with the introduction of this compound as a preservative. MI was considered to be the most important sensitizer in the mixture, and in 2013 it was designated as the “contact allergen of the year” by the American Contact Dermatitis Society (ACDS) (26). In Europe, the prevalence of contact allergies due to MCI/MI and, mostly, MI, has greatly increased between 2010 and 2013, mainly because of the extensive presence of MI in cosmetic products (26, 27). For this reason, the concentration of the mixture MCI/MI was restricted both in the European Union and the United States. In 1992, the Cosmetic Ingredients Review Expert Panel concluded that the MCI/MI blend was considered safe in rinse-off products at a concentration not to exceed 15 ppm (parts per million; 0.0015%) and in leave-on products at a concentration not to exceed 7.5 ppm (and these are the limits set today in the US). In July 2017, the European Committee, because of the increasing prevalence of sensitization to MI, modified regulation No. 1223/2009 of the European Parliament and of the Council on cosmetic products according to indications formulated by the Scientific Committee on Consumer Safety on 15 December 2015. Specifically, for cosmetic products to be rinsed, a maximum concentration of 0.0015% (15 ppm) of MI was established to be safe for consumers. From 27 January 2018, only cosmetic products, according to this regulation, can be placed on the market in the European Union (29).

Finally, we report that a few cases of co-sensitization to MCI/MI and imidazoles/nitroimidazoles are described, probably related to a similar spatial geometry between these molecules (30, 31).

### 5.3. Fragrances

The prevalence of fragrance sensitivity is estimated between 0.7 and 2.6%, with a positive patch test reaction rate ranging from 5 to 11% (32). Fragrances include a series of many compounds, usually synthetic. In addition to perfumes, they are contained in cologne, eau de toilette, and aftershave. They are also present in cosmetics (for skin care, nails, hair, and eyes), toothpaste, sun creams, and adult and child cleansing products, including wet wipes and insect repellents. Fragrances can also be found in household products such as dish and clothing detergents, softeners, environmental deodorants, waxes, furniture polish, and utensils. They can also be used in some food flavors. From a diagnostic point of view, fragrances are usually tested as mixes. Fragrance Mix I contains eugenol, isoeugenol, cinnamic alcohol, cinnamic aldehyde, amylcinnamaldehyde, geraniol, and hydroxytronellal, absolute oak moss. Fragrance Mix II includes lyral, citral, farnesol, citronellol, coumarin, and hexylcinnamaldehyde. Lyral is also tested by itself and Balsam of Peru (33).

Balsam of Peru, a tree resin derived from *Myroxylon pereirae*, is still considered a marker for the detection of a relevant number of fragrance allergies (34). This resin’s characteristic sweet and vanilla smell comprises various fragrant components. It is estimated that nearly 50% of patients with a fragrance allergy will react to this allergen.

During testing fragrances, clinicians must remember that patients can cross-react with essential oils that should be tested separately using the patient’s products and that subjects sensitized with colophony and propolis can show cross-reactions with fragrances in patch testing.

### 5.4. Formaldehyde

The prevalence of contact sensitization to formaldehyde is reported to be between 0.97 and 2.3% (35). Formaldehyde is a colorless liquid with a pungent odor that is generally used as a disinfectant and is widely used in healthcare and domestic environments. Particularly, it is used for the synthesis of many resins, including urea-formaldehyde, melamine-formaldehyde resins, and cyclized urea derivatives that can be used for the treatment of some fabrics (cotton, cotton/polyester, wrinkle-resistant linen) for various purposes (anti-crease finishing, a fixer for dyes, anti-mold, binders for prints) and release formaldehyde in variable quantities according to the type of treatment to which the fabric has been subjected. Repeated washing of personal clothing and furnishing fabrics at 60 ° C reduces the possibility of contact with formaldehyde (36). In the industrial sector, it is present in urea-formaldehyde resins and foams for insulation, in the textile industry, in the wood industry (pressed wood: chipboard, plywood), in the engineering industry (coolants), and in inks, paints, toners, adhesives, waxes, and enamels. Formaldehyde is also used in histopathology laboratories, in the health sector as a fixator for histological pieces, and in photographic laboratories. Formaldehyde can also be released from some preservatives (2-bromo-2-nitropropane-1,3-diol, Diazolidinyl urea, DMDM hydantoin, imidazolidinyl urea, Quaternium15), which may be present in cosmetics (face creams, mascara, foundation, deodorants, shampoos, hair conditioners, nail hardeners, toothpaste) and topical medications to prevent contamination and deterioration by microorganisms (35, 36).

### 5.5. *P*-phenylenediamine

1,4-phenylenediamine, or paraphenylenediamine (PPD), is a toxic and irritating aromatic diamine currently used as a darkening agent in hair dyes and temporary tattoos and as a monomer in the production of Kevlar, a heat-resistant and strong synthetic fiber. A recent cross-sectional study on a population of five Europeans showed a prevalence of PPD contact allergy of 0.8% (37).

Hair dyes represent the main source of contact sensitization to this allergen. PPD is also often used in temporary tattoos (such as henna tattoos) to intensify their color and increase their fixation on the skin. It may also be present in dark-colored cosmetics, black gums, developing liquids for photography and lithographic tools, dark fabrics, and clothing.

The concentration of PPD in temporary tattoos was found to be up to about 15%, which is more than double that allowed in permanent dyes. Moreover, the occlusion of the tattoo with gloves, stockings, and plasters used for fixing the henna to the skin increases the penetration of the allergen, promoting the sensitization phase. The use of PPD is restricted by EU legislation due to its high sensitizing power. In 1976, the maximum allowed PPD concentration in hair dyes was 6%. Subsequently, in 2009, after being mixed with the oxidizing base to be applied directly to the outer hair, it was reduced to 2%. In the United States, there is no regulation about this issue, while the current European regulations establish that PPD should not be applied directly to the skin, eyebrows, or eyelashes. Therefore, its use is prohibited for both temporary and permanent tattoos (38).

Cross-reactions can occur between paraphenylenediamine and substances of the “para” group, such as:

- azo dyes (used to color fabrics)
- benzocaine (local anesthetic)
- *p*-aminodiphenylmethane (the antioxidant in the rubber manufacturing process)
- *p*-toluene diamine and aminophenols (hair dyes)
- *p*-aminobenzoic acid or PABA (sunscreen)

Patients sensitized to paraphenylenediamine may also have exacerbations of dermatitis following the intake of drugs such as sulfamicides, benzothiazide diuretics, furosemide, sulfonylureas (oral hypoglycemic agents), and *p*-aminosalicylic acid (39).

### 5.6. Emergent allergens

Developing new technologies, chemicals, and personal products leads to exposure to new potential allergens.

#### 5.6.1. Sorbitan sesquioleate

Sorbitan sesquiplane is an oil-soluble surfactant derived from sorbitol and oleic acid. It is used in a large variety of products, including skincare and cleansing products, moisturizers, and eye and face makeup, as an emulsifier and moisturizing agent. It is also used in topical corticosteroids, antibiotics, and antifungals, primarily as an emulsifier. Sorbitan sesquiplane is sometimes used as a food additive and can be found in some veterinary products and household items (40). A recent Italian multicenter SIDAPA (Società Italiana Dermatologia Allergologica Professionale Ambientale) study has shown that the prevalence of positivity to sorbitan sesquiplane in a 1-year period of consecutive series of 0.5% meets the threshold for inclusion in the baseline series (41).

#### 5.6.2. Acrylates and methacrylates

Acrylates and methacrylates are reactive monomers that polymerize into polymer plastics. They are well known as strong contact sensitizers, both in occupational (nail art technicians, dentists, dental technicians) and non-occupational (dental, orthopedic prostheses, medical devices, such as electrocardiography electrodes and glucose sensors, artificial nails, wound care products) settings (42–47). Nowadays, nail cosmetics (artificial nails and long-lasting, UV-cured, acrylate-based nail polish) represent the main exposure source to (meth) acrylates contact allergy (43). ACD due to nail (meth) acrylates is mainly reported in adults (48), even though recent cases have been described in adolescents and children (49, 50). In Italy, we observed that nail (meth) acrylates were associated with a positive patch test to 2-Hydroxyethyl methacrylate (2-HEMA), proposed as a marker of contact allergy to (meth) acrylates, in 80.5% of patients, especially in consumers (72.7%) (43). In 2018, the British Society for Cutaneous Allergy recommended the addition of 2-HEMA 2% pet to the baseline patch test series and ESCD in 2019 (1, 7). Since 2016, 2-HEMA 2% pet has been included in the SIDAPA baseline and serves as a marker for contact sensitization to acrylates and methacrylates (51).

#### 5.6.3. Topical medications

Allergic contact dermatitis can also occur with topical medications (52, 53), such as topical antibiotics (54–56), antiacne drugs (57), anesthetics (58, 59), antihistamines (60, 61), testosterone and estrogen from the transdermal therapeutic system (52), antimycotics (62–64), sunscreens (65–67), NSAIDs (68, 69) corticosteroids (CS) (52, 70).

CSs were first used in the 1950s in the first case of ACD, and nowadays, they represent the cornerstone of the treatment of ACD, but they can paradoxically cause ACD themselves (52). CS hypersensitivity is a challenging issue, especially in choosing an alternative and safe topical and systemic CS, due to the high frequency of cross-reactions among CS groups (71–74). The molecular structure and configuration of CSs were studied and classified by Baeck (71) into three different groups. Cross-reactions between CSs occur more commonly within each group, but they can also involve CSs belonging to different groups and between both topical and systemic CSs (75). Baeck’s clusters do not include deflazacort, which presents a 16α-17α-2 methyloxazolinic ring, which distinguishes it from other CSs.

Nevertheless, even if deflazacort has been considered a safe alternative in patients with multiple sensitizations to CSs (76), recent literature data suggest that cross-reactions may also occur between this molecule and other CSs (75, 77, 78). Budesonide can be considered a marker of CSs allergy because it has proven capable of detecting sensitization to both topical and systemic CSs (71, 79). A recent case series found a prevalence of 60.9% positive patch tests for at least one systemic CS in patients with documented sensitization to budesonide (80). The main source of contact with topical CSs is a topical medication used for the treatment of inflammatory skin diseases (creams and ointments), but there are also topical drugs that can be used over mucous membranes (eyedrops) and upper airways (aerosols and sprays). Diagnosing ACD in topical CSs requires a complete diagnostic approach based on excluding cross-reactivity between topical and systemic CSs.

ACD by topical medications can also be induced by excipients or preservatives used in topical treatment products or personal care products (81), such as propylene glycol (named “Allergen of the Year” by the American Contact Dermatitis Society in 2018) (82), sorbitan sesquiplane, lanolin or wool alcohols, parabens, and cetyl stearyl alcohol (81, 83–85). Moreover, contaminants present in final topical treatments and cosmetic products, as well as medical devices, can induce contact allergies (81, 86–91).

#### 5.6.4. Textiles

The prevalence of ACD induced by textiles, both in occupational and non-occupational settings, seems to be increasing, probably because of the introduction of new textile manufacturing techniques (92). Among the textile allergens, disperse dyes are the most common sensitizers, followed by formaldehyde and resins (92–94). In 2015, a textile dye mix (TDM) of 6.6% containing eight dispersed days was included in the baseline patch test series by ESCD. In Europe, it has been estimated that the prevalence of TDM is 6.6% positive on a patch test, ranging from 2.1 to 6.9%. In Italy, a recent 2-year multicenter study revealed a prevalence of sensitization to TDM of 1.5%, supporting the importance of testing this allergen in the baseline series (94).

## 6. Diagnosis

Medical history provides the basis for a complete allergological workup. It may include information about a patient’s job, hobbies, drugs taken, topical cosmetics or medications applied, and textiles worn. A physical examination with an evaluation of morphology and localization of skin/mucous lesions is also required. This initial approach is necessary to hypothesize about or identify a possible suspect sensitizer.

A patch test is considered the gold standard for diagnosing ACD. This skin test aims to replicate the elicitation phase of type IV hypersensitivity by applying under occlusion a specific allergen to the skin with a standardized procedure (95).

So long as a correct evaluation of culprit allergens and their clinical relevance is difficult (also for a skilled dermatologist), the “baseline series,” comprising the most common allergens in different countries, was introduced. Moreover, when baseline series is not sufficient according to the patient’s clinical history and allergen exposure, additional patch test series or allergens must be tested. Criteria for including an allergen in a baseline series are a proportion of confirmed contact allergy to the compound over 0.5–1% in a number of consecutive patches tested in patients with suspected ACD (95, 96).

Allergens are dispersed in petrolatum, or sometimes in water or ethanol, and applied to the skin in small chambers (e.g., Finn Chambers®, Van der Bend®, or IQ Ultra®) under occlusion, mainly on the upper back. Before applying patch tests, a clinician must verify some conditions that contraindicate the test procedure, specifically the presence of a severe or generalized dermatitis or an upper back dermatitis, a systemic immunosuppressive treatment (e.g., prednisolone >10 mg daily), the recent application of topical corticosteroids, which should be stopped at least 7 days before testing, or recent ultraviolet exposure of the test area.

According to the Italian Guidelines for Patch Testing (95), the patch test chambers must be removed on day 2 (D2), 48 h after application at D0. A first reading is performed at D2 15–60 min after detachment of chambers, and the second one is mandatory at D3 or D4. A third reading, between D5 and D10, is necessary for some allergens, such as corticosteroids and aminoglycoside antibiotics.

Inspection and palpation of the application site of the allergen must be performed at readings. The morphology of the reaction is reported as “+,” “++,” or “+++,” considering the presence of erythema, infiltration, papules, vesicles, or bullae (Table 2). Some substances may induce an irritant reaction that has to be interpreted as false positivity, especially if the application site shows sharp-edged margins of erythema and fine surface wrinkling. After the patch test readings, findings must be evaluated considering the patient’s history, environment, and clinical course to clarify the relevance of the reaction (95, 96).

**Table 2:** Reading criteria of the ICDRG, adapted from the study by Stingeni et al. (95).

Symbol	Morphology	Assessment
−	No reaction	Negative reaction
?+	Faint erythema only	Doubtful reaction
+	Erythema, infiltration, possibly papules	Weak positive reaction
++	Erythema, infiltration, papules, vescicles	Strong positive reaction
+++	Intense erythema, infiltrate, coalescing vescicles	Extreme positive reaction
IR	Various morphology, e.g., soap effect, bulla, necrosis	Irritant reaction

Another technique is the Repeated Open Application Test (ROAT), developed by Hannuksela et al. (97). This method aims at eliciting ACD in a standardized area by repeated application of a commercial product, similar to a common-use application. This *in vivo* test is performed by applying the suspected product twice a day for up to 2 weeks to a 3 × 3 to 5 × 5 cm area localized on the volar aspect of the antecubital area. The appearance of an eczematous reaction at the site of ROAT, usually a few days after the beginning of the test, must be considered a positive. However, the absence of a reaction after the test does not exclude a contact allergy. In some cases, if the history is particularly suspicious, the test can be prolonged for 3–4 weeks. The semi-open test is another diagnostic technique that can be useful for irritant products (e.g., shampoos, detergents, and cosmetics). It is performed by applying a small amount of suspect allergen with a cotton swab to a 1 cm^2^ area and letting it dry. If no signs of contact dermatitis are observed after 20–30 min, it may be covered with tape. Readings are performed the same way as for patch tests. An open test may also be performed, without occlusion, often as a first diagnostic step with the patient’s own products when history is doubtful.

If photoallergic contact dermatitis is suspected, a photopatch test should be performed. A double set of allergens is applied on the back of the patient, and at D2, one of the two sets is irradiated with 5 J/cm^2^ of UVA, covering the other set from light. Readings are performed before and after UV irradiation and then at D4. A positive reaction to the photo patch test is a positive (+ to +++) reaction at the irradiated site with no reaction at the non-irradiated site, while positivity in both sets is to be interpreted as ACD (95, 96).

## 7. Treatment

Avoiding contact with the identified allergen is crucial to treating and preventing ACD. Moreover, it is essential to provide comprehensive information to patients about the allergen, especially concerning sources, ways of exposure, and the use of alternative substances.

Some websites can generate lists of products that are safe to use and products that should be avoided, even to help the patient keep in mind possible cross-reactions such as SkinSAFE (98) and CAMP (Contact Allergen Management Program) (99).

Correct management of ACD is crucial to preventing dermatitis from becoming a chronic condition, especially if dermatitis is localized in relevant sites, such as the face or hands, with a high disease burden and impairment of quality of life (100–102).

The treatment of ACD is also based on a short course of mid- to high-dose topical corticosteroids (Table 3) (103). If ACD is severe, systemic corticosteroids can be used, as well as oral antihistamines, in case of intense itch. If a steroid-free treatment is preferred to avoid side effects such as skin atrophy and telangiectasias, topical calcineurin inhibitors can be applied, even for long-term therapies. In severe or relapsing ACD, it can be reasonable to opt for phototherapy with immunosuppressive agents such as cyclosporine, mycophenolate mofetil, and azathioprine (1–3, 6).

**Table 3:** Potency classification of topical corticosteroids, adapted from the study by Parikh et al. (103).

Potency	Class	Topical corticosteroid	Formulation
Ultrahigh	1	Clobetasol propionate	Cream, 0.05%
High	2	Betamethasone dipropionate	Ointment, 0.05%
		Fluocinonide	Cream, ointment or gel, 0.05%
	3	Betamethasone dipropionate	Cream, 0.05%
		Betamethasone valerate	Ointment, 0.1%
		Triamcinolone acetonide	Ointment, 0.1%
Moderate	4	Desoximetasone	Cream, 0.05%
		Fluocinolone acetonide	Ointment, 0.025%
		Hydrocortisone valerate	Ointment, 0.2%
		Triamcinolone acetonide	Cream, 0.1%
	5	Betamethasone dipropionate	Lotion, 0.02%
		Betamethasone valerate	Cream, 0.1%
		Fluocinolone acetonide	Cream, 0.025%
		Hydrocortisone butyrate	Cream, 0.1%
		Hydrocortisone valerate	Cream, 0.2%
		Triamcinolone acetonide	Lotion, 0.1%
Low	6	Betamethasone valerate	Lotion, 0.05%
		Desonide	Cream, 0.05%
		Fluocinolone acetonide	Solution, 0.01%
	7	Dexamethasone sodium phosphate	Cream, 0.1%
		Hydrocortisone acetate	Cream, 1%
		Methylprednisolone acetate	Cream, 0.25%

Recent evidence demonstrates that biologics can represent a possible therapeutic alternative to ACD.

In a recent review, dupilumab, a monoclonal antibody approved for atopic dermatitis (AD) and other conditions, such as asthma, was studied in patients affected by AD and ACD, showing that these patients had a similar decrease in BSA, IGA, and pruritus compared with a patient affected by AD but a negative PT in treatment with dupilumab (104). These observations are supported by the most recent studies showing that the cytokine response in ACD is hapten-specific with both Th1 and Th2 involvement (17, 105).

Moreover, preliminary evidence suggests that atopic patients with ACD treated with dupilumab do not significantly experience the peculiar fluctuation in eosinophil counts that is typically observed during the first months of therapy (106).

Finally, Bangsgaard et al. (107) reported using ustekinumab, a monoclonal antibody blocking IL-12 and IL-23, to treat five patients affected by ACD and polysensitized (positive PT to more than three allergens), showing no efficacy of the drug.

## Author contributions

MT and LS contributed to conception and design of the study, and wrote the first draft of the manuscript. KH, LB, and CS wrote sections of the manuscript. All authors contributed to manuscript revision, read, and approved the submitted version.

## Conflict of interest

The authors declare that the research was conducted in the absence of any commercial or financial relationships that could be construed as a potential conflict of interest.

## Publisher’s note

All claims expressed in this article are solely those of the authors and do not necessarily represent those of their affiliated organizations, or those of the publisher, the editors and the reviewers. Any product that may be evaluated in this article, or claim that may be made by its manufacturer, is not guaranteed or endorsed by the publisher.

## References

[1] Elmas ÖF Akdeniz N Atasoy M Karadag AS. Contact dermatitis: a great imitator. Clin Dermatol. (2020) 38:176–92. doi: 10.1016/j.clindermatol.2019.10.003, PMID: 32513398

[2] Rashid RS Shim TN. Contact dermatitis. BMJ. (2016) 30:353. doi: 10.1136/bmj.i3299 27364956

[3] Usatine RP Riojas M. Diagnosis and management of contact dermatitis. Am Fam Physician. (2010) 82:249–55. PMID: 20672788

[4] Ale IS Maibach HI. Irritant contact dermatitis. Rev Environ Health. (2014) 29:195–206. doi: 10.1515/reveh-2014-0060 25274939

[5] Slodownik D Lee A Nixon R. Irritant contact dermatitis: a review. Australas J Dermatol. (2008) 49:1–11. doi: 10.1111/j.1440-0960.2007.00409.x 18186838

[6] Lachapelle JM. Allergic contact dermatitis: clinical aspects. Rev Environ Health. (2014) 29:185–94. doi: 10.1515/reveh-2014-0055 25241725

[7] Statescu L Branisteanu D Dobre C Solovastru LG Vasilca A Petrescu Z. Contact dermatitis – epidemiological study. Maedica (Bucur). (2011) 6:277–81. PMID: 22879841 PMC3391944

[8] Hollins LC Flamm A. Occupational contact dermatitis: evaluation and management considerations. Dermatol Clin. (2020) 38:329–38. doi: 10.1016/j.det.2020.02.001 32475511

[9] Alinaghi F Bennike NH Egeberg A Thyssen JP Johansen JD. Prevalence of contact allergy in the general population: a systematic review and meta-analysis. Contact Dermatitis. (2019) 80:77–85. doi: 10.1111/cod.13119, PMID: 30370565

[10] Kostner L Anzengruber F Guillod C Recher M Schmid-Grendelmeier P Navarini AA. Allergic contact dermatitis. Immunol Allergy Clin N Am. (2017) 37:141–52. doi: 10.1016/j.iac.2016.08.014 27886903

[11] Botto N Raffi J Trivedi M Ramirez F Allen IE Chren MM. Validating a quality-of-life instrument for allergic contact dermatitis. Dermatitis. (2019) 30:300–5. doi: 10.1097/DER.0000000000000515 31524758

[12] Nassau S Fonacier L. Allergic contact dermatitis. Med Clin North Am. (2020) 104:61–76. doi: 10.1016/j.mcna.2019.08.012 31757238

[13] Borok J Matiz C Goldenberg A Jacob SE. Contact dermatitis in atopic dermatitis children-past, present, and future. Clin Rev Allergy Immunol. (2019) 56:86–98. doi: 10.1007/s12016-018-8711-2, PMID: 30225535

[14] Teo Y McFadden JP White IR Lynch M Banerjee P. Allergic contact dermatitis in atopic individuals: results of a 30-year retrospective study. Contact Dermatitis. (2019) 81:409–16. doi: 10.1111/cod.13363, PMID: 31347185

[15] Hamann CR Hamann D Egeberg A Johansen JD Silverberg J Thyssen JP. Association between atopic dermatitis and contact sensitization: a systematic review and meta-analysis. J Am Acad Dermatol. (2017) 77:70–8. doi: 10.1016/j.jaad.2017.02.001, PMID: 28392290

[16] Brar KK. A review of contact dermatitis. Ann Allergy Asthma Immunol. (2021) 126:32–9. doi: 10.1016/j.anai.2020.10.003 33091591

[17] Dhingra N Shemer A Correa da Rosa J Rozenblit M Fuentes-Duculan J Gittler JK. Molecular profiling of contact dermatitis skin identifies allergen-dependent differences in immune response. J Allergy Clin Immunol. (2014) 134:362–72. doi: 10.1016/j.jaci.2014.03.009, PMID: 24768652

[18] Angelini G Vena GA. Dermatologia professionale e ambientale. eds. Bonamonte D Curatoli G Filotico R Foti C Grandolfo M Mastrolonard M, Brescia (IT): ISED (1997).

[19] Santos R Goossens A. An update on airborne contact dermatitis: 2001-2006. Contact Dermatitis. (2007) 57:353–60. doi: 10.1111/j.1600-0536.2007.01233.x, PMID: 17988283

[20] Saurat JH Lipsker D Borradori L Lachapelle JM. Dermatologie et infections sexuellement transmissibles. 6th ed. ed. Calzavara Pinton P. Issy les Moulineaux (FR): Elsevier Masson SAS (2017).

[21] Peltonen L. Nickel sensitivity in the general population. Contact Dermatitis. (1979) 5:27–32. doi: 10.1111/j.1600-0536.1979.tb05531.x 421456

[22] Uter W Amario-Hita JC Balato A Ballmer-Weber B Bauer A Belloni Fortina A. European surveillance system on contact allergies (ESSCA): results with the European baseline series, 2013/14. J Eur Acad Dermatol Venereol. (2017) 31:1516–25. doi: 10.1111/jdv.14423, PMID: 28627111

[23] Kornik R Zug KA. Nickel. Dermatitis. (2008) 19:3–8. doi: 10.2310/6620.2008.07082 18346389

[24] Tramontana M Bianchi L Hansel K Agostinelli D Stingeni L. Nickel allergy: epidemiology, pathomechanism, clinical patterns, treatment and prevention programs. Endocr Metab Immune Disord Drug Targets. (2020) 20:992–1002. doi: 10.2174/1871530320666200128141900, PMID: 31994473

[25] Pizzutelli S. Systemic nickel hypersensitivity and diet: myth or reality? Eur Ann Allergy Clin Immunol. (2011) 43:5–18. PMID: 21409856

[26] Castanedo-Tardana MP Zug KA. Methylisothiazolinone. Dermatitis. (2013) 24:2–6. doi: 10.1097/DER.0b013e31827edc73 23340392

[27] Puangpet P Chawarung A JP MF. Methylchloroisothiazolinone/methylisothiazolinone and methylisothiazolinone allergy. Dermatitis. (2015) 31:1–3. doi: 10.1097/DER.0000000000000105 25757082

[28] Stingeni L Bianchi L Foti C Romita P Rigano L Hansel K. An Italian multicenter study on methylchloroisothiazolinone/methylisothiazolinone contact sensitivity: understanding the structure-activity relationship. Contact Dermatitis. (2018) 78:297–9. doi: 10.1111/cod.12935, PMID: 29527728

[29] REGOLAMENTO (UE) 2017/1224 DELLA COMMISSIONE. del 6 luglio (2017). Issy les Moulineaux (FR). Available at: https://eur-lex.europa.eu/legal-content/IT/TXT/PDF/?uri=CELEX:32017R1224&from=EN

[30] Nasir S Goldsmith P. Anogenital allergic contact dermatitis caused by methylchloroisothiazolinone, methylisothiazolinone and topical clotrimazole with subsequent generalized exanthem triggered by oral fluconazole. Contact Dermatitis. (2016) 74:296–7. doi: 10.1111/cod.12513, PMID: 27040872

[31] Stingeni L Rigano L Lionetti N Bianchi L Tramontana M Foti C. Sensitivity to imidazoles/nitroimidazoles in subjects sensitized to methylchloroisothiazolinone/methylisothiazolinone: a simple coincidence? Contact Dermatitis. (2019) 80:181–3. doi: 10.1111/cod.13158, PMID: 30468019

[32] Reeder MJ. Allergic contact dermatitis to fragrances. Dermatol Clin. (2020) 38:371–7. doi: 10.1016/j.det.2020.02.009, PMID: 32475515

[33] Kumar M Devi A Sharma M Kaur P Mandal UK. Review on perfume and present status of its associated allergens. J Cosmet Dermatol. (2021) 20:391–9. doi: 10.1111/jocd.13507, PMID: 32445606

[34] Guarneri F Corazza M Stingeni L Patruno C Napolitano M Pigatto PDM. *Myroxylon pereirae* (balsam of Peru): still worth testing? Contact Dermatitis. (2021) 85:269–73. doi: 10.1111/cod.13839, PMID: 33748955 PMC8453940

[35] Fasth IM Ulrich NH Johansen JD. Ten-year trends in contact allergy to formaldehyde and formaldehyde-releasers. Contact Dermatitis. (2018) 79:263–9. doi: 10.1111/cod.13052, PMID: 30079600

[36] Pontén A Bruze M. Formaldehyde. Dermatitis. (2015) 26:3–6. doi: 10.1097/DER.0000000000000075 25581665

[37] Diepgen TL Naldi L Bruze M Cazzaniga S Schuttelaar ML Elsner P. Prevalence of contact allergy to *p*-phenylenediamine in the European general population. J Invest Dermatol. (2016) 136:409–15. doi: 10.1016/j.jid.2015.10.064, PMID: 26802237

[38] Encabo Durán B Romero-Pérez D Silvestre Salvador JF. Allergic contact dermatitis due to paraphenylenediamine: an update. *Actas Dermosifiliogr* (Engl Ed). (2018) 109:602–9. doi: 10.1016/j.ad.2017.12.007, PMID: 29496197

[39] Boyd AH Zhang AJ Hylwa SA. Seeing double: allergic contact dermatitis to Para-amino compounds. Dermatitis. (2018) 29:285–6. doi: 10.1097/DER.0000000000000399, PMID: 30179973

[40] Asarch A Scheinman PL. Sorbitan sesquioleate: an emerging contact allergen. Dermatitis. (2008) 19:339–41. doi: 10.2310/6620.2008.08029, PMID: 19134439

[41] Stingeni L Tramontana M Bianchi L Foti C Romita P Patruno C. Patch test with sorbitan sesquioleate in Italian consecutive patients: a 1-year multicenter SIDAPA study. Contact Dermatitis. (2019) 81:454–6. doi: 10.1111/cod.13359, PMID: 31328272

[42] Voller LM Warshaw EM. Acrylates: new sources and new allergens. Clin Exp Dermatol. (2020) 45:277–83. doi: 10.1111/ced.14093, PMID: 31565812

[43] Stingeni L Tramontana M Bianchi L Foti C Patruno C Gallo R. Contact sensitivity to 2-hydroxyethyl methacrylate in consecutive patients: a 1-year multicenter SIDAPA study. Contact Dermatitis. (2019) 81:216–8. doi: 10.1111/cod.13278, PMID: 30916788

[44] Tramontana M Hansel K Bianchi L Foti C Romita P Stingeni L. Occupational allergic contact dermatitis from a glue: concomitant sensitivity to "declared" isothiazolinones and "undeclared" (meth)acrylates. Contact Dermatitis. (2020) 83:150–2. doi: 10.1111/cod.13569, PMID: 32311096

[45] Hansel K Tramontana M Bianchi L Brozzi J Stingeni L. Allergic contact stomatitis to dental prosthesis due to acrylic monomers with cross-reactivity to 2-hydroxyethyl methacrylate. Dermatitis. (2020) 31:e28–30. doi: 10.1097/DER.0000000000000571, PMID: 32049714

[46] Hansel K Tramontana M Bianchi L Cerulli E Patruno C Napolitano M. Contact sensitivity to electrocardiogram electrodes due to acrylic acid: a rare cause of medical device allergy. Contact Dermatitis. (2020) 82:118–21. doi: 10.1111/cod.13403, PMID: 31566738

[47] Tramontana M Hansel K Bianchi L Agostinelli D Stingeni L. Allergic contact dermatitis caused by a glucose monitoring system: an emerging side-effect of diabetes medical devices. J Eur Acad Dermatol Venereol. (2020) 34:e223–5. doi: 10.1111/jdv.16178, PMID: 31904884

[48] Dahlin J Berne B Dunér K Hosseiny S Matura M Nyman G. Several cases of undesirable effects caused by methacrylate ultraviolet-curing nail polish for non-professional use. Contact Dermatitis. (2016) 75:151–6. doi: 10.1111/cod.12608, PMID: 27230069

[49] Romita P Foti C Barlusconi C Hansel K Tramontana M Stingeni L. Contact allergy to (meth)acrylates in gel nail polish in a child: an emerging risk for children. Contact Dermatitis. (2020) 83:39–40. doi: 10.1111/cod.13503, PMID: 32100300

[50] Tramontana M Hansel K Bianchi L Marietti R Stingeni L. Use of self-applied sculptured gel nails may increase the risk of allergy to (meth)acrylates in children and adolescents. J Eur Acad Dermatol Venereol. (2021) 35:e765–7. doi: 10.1111/jdv.17429, PMID: 34062011

[51] Hansel K Foti C Nettis E Lopalco A Tramontana M Bianchi L. Acrylate and methacrylate allergy: when is patch testing with acrylic acid recommended? Contact Dermatitis. (2020) 82:231–3. doi: 10.1111/cod.13440, PMID: 31777076

[52] Nguyen HL Yiannias JA. Contact dermatitis to medications and skin products. Clin Rev Allergy Immunol. (2019) 56:41–59. doi: 10.1007/s12016-018-8705-0 30145645

[53] Thaiwat S Ubol T. Allergic contact dermatitis to topical medicaments: revisited. Asian Pac J Allergy Immunol. (2020). doi: 10.12932/AP-180820-0944, PMID: 33274954

[54] Gehrig KA Warshaw EM. Allergic contact dermatitis to topical antibiotics: epidemiology, responsible allergens, and management. J Am Acad Dermatol. (2008) 58:1–21. doi: 10.1016/j.jaad.2007.07.050, PMID: 18158924

[55] Voller LM Kullberg SA Warshaw EM. Axillary allergic contact dermatitis to topical clindamycin. Contact Dermatitis. (2020) 82:313–4. doi: 10.1111/cod.13465, PMID: 31907935

[56] Romita P Stingeni L Hansel K Ettorre G Bosco A Ambrogio F. Allergic contact dermatitis caused by chloramphenicol with prurigo nodularis-like spreading. Contact Dermatitis. (2019) 80:251–2. doi: 10.1111/cod.13187, PMID: 30485440

[57] Veraldi S Brena M Barbareschi M. Allergic contact dermatitis caused by topical antiacne drugs. Expert Rev Clin Pharmacol. (2015) 8:377–81. doi: 10.1586/17512433.2015.1046839, PMID: 25982754

[58] Kimyon RS Schlarbaum JP Liou YL Hylwa SA Warshaw EM. Allergic contact dermatitis to pramoxine (pramocaine). Dermatitis. (2021) 32:32–7. doi: 10.1097/DER.0000000000000606, PMID: 32404620

[59] Hansel K Sensini C Caposciutti P Marietti R Tramontana M Bianchi L. Systemic allergic dermatitis during tetracaine hydrochloride patch testing. Contact Dermatitis. (2021) 85:596–8. doi: 10.1111/cod.13921, PMID: 34180549

[60] Romita P Foti C Stingeni L. Photoallergy to promazine hydrochloride. Contact Dermatitis. (2017) 77:182–3. doi: 10.1111/cod.12797, PMID: 28766806

[61] Romita P Stingeni L Barlusconi C Hansel K Foti C. Allergic contact dermatitis in response to ketotifen fumarate contained in eye drops. Contact Dermatitis. (2020) 83:35–7. doi: 10.1111/cod.13497, PMID: 32064627

[62] Guidetti MS Vincenzi C Guerra L Tosti A. Contact dermatitis due to imidazole antimycotics. Contact Dermatitis. (1995) 33:282. doi: 10.1111/j.1600-0536.1995.tb00495.x, PMID: 8654095

[63] Romita P Stingeni L Hansel K De Prezzo S Ambrogio F Bonamonte D. Allergic contact dermatitis caused by amorolfine in a nail lacquer. Contact Dermatitis. (2019) 81:407–8. doi: 10.1111/cod.13356, PMID: 31314139

[64] Pérez-Mesonero R Schneller-Pavelescu L Ochando-Ibernón G Vergara-Sánchez A Sánchez-Herreros C Martín-Alcalde E. Is tioconazole contact dermatitis still a concern? Bringing allergic contact dermatitis caused by topical tioconazole back into the spotlight. Contact Dermatitis. (2019) 80:168–9. doi: 10.1111/cod.13146, PMID: 30411354

[65] Tracz ES Sommerlund M Bregnhøj A. Pustular allergic contact dermatitis caused by a sunscreen. Contact Dermatitis. (2020) 83:328–9. doi: 10.1111/cod.13621, PMID: 32510629

[66] Badaoui A Vergez M Soria A. Allergic contact dermatitis from oleoyl tyrosine in a sunscreen. Contact Dermatitis. (2021) 85:255–6. doi: 10.1111/cod.13835 33740263

[67] Romita P Foti C Hansel K Stingeni L. Photo-contact allergy to octocrylene: a decreasing trend? Contact Dermatitis. (2018) 78:224–5. doi: 10.1111/cod.12904, PMID: 29430706

[68] Gulin SJ Chiriac A. Diclofenac-induced allergic contact dermatitis: a series of four patients. Drug Saf Case Rep. (2016) 3:15. doi: 10.1007/s40800-016-0039-3, PMID: 27882527 PMC5120621

[69] Romita P Barlusconi C Mercurio CS Hansel K Stingeni L Foti C. Photoallergic contact cheilitis from benzydamine hydrochloride contained in a mouthwash. Contact Dermatitis. (2020) 83:130–2. doi: 10.1111/cod.13541, PMID: 32232858

[70] Stingeni L Bianchi L Tramontana M Pigatto PD Patruno C Corazza M. Skin tests in the diagnosis of adverse drug reactions. G Ital Dermatol Venereol. (2020) 155:602–21. doi: 10.23736/S0392-0488.20.06698-5 32938165

[71] Baeck M Chemelle JA Goossens A Nicolas JF Terreux R. Corticosteroid cross-reactivity: clinical and molecular modeling tools. Allergy. (2011) 66:1367–74. doi: 10.1111/j.1398-9995.2011.02666.x, PMID: 21671945

[72] Hansel K Marietti R Bianchi L Tramontana M Foti C Romita P. Cross-reactions to systemic corticosteroids in patients contact sensitized to budesonide. Contact Dermatitis. (2020) 83:321–4. doi: 10.1111/cod.13597, PMID: 32386100

[73] Coopman S Degreef H Dooms-Goossens A. Identification of cross-reaction patterns in allergic contact dermatitis from topical corticosteroids. Br J Dermatol. (1989) 121:27–34. doi: 10.1111/j.1365-2133.1989.tb01396.x, PMID: 2757954

[74] Bianchi L Marietti R Tramontana M Hansel K Stingeni L. Systemic allergic dermatitis from intra-articular triamcinolone acetonide: report of two cases with unusual clinical manifestations. Contact Dermatitis. (2021) 84:54–6. doi: 10.1111/cod.13667, PMID: 32677172

[75] Bianchi L Hansel K Antonelli E Bellini V Rigano L Stingeni L. Deflazacort hypersensitivity: a difficult-to-manage case of systemic allergic dermatitis and literature review. Contact Dermatitis. (2016) 75:54–6. doi: 10.1111/cod.12555, PMID: 27264291

[76] Ventura MT Calogiuri GF Matino MG Dagnello M Buquicchio R Foti C. Alternative glucocorticoids for use in case of adverse reaction to systemic glucocorticoids: a study on 10 patients. Br J Dermatol. (2003) 148:139–41. doi: 10.1046/j.1365-2133.2003.05061.x 12534608

[77] Navarro Pulido AM Orta JC Buzo G. Delayed hypersensitivity to deflazacort. Allergy. (1996) 51:441–2. doi: 10.1111/j.1398-9995.1996.tb04646.x, PMID: 8837672

[78] Garcia-Bravo B Repiso JB Camacho F. Systemic contact dermatitis due to deflazacort. Contact Dermatitis. (2000) 43:359–60. doi: 10.1034/j.1600-0536.2000.043006359.x, PMID: 11140387

[79] Hansel K Marietti R Tramontana M Bianchi L Rigano L Lionetti N. Sensitization/tolerance profile of betamethasone sodium phosphate in patients with contact allergy to budesonide. Dermatol Ther. (2020) 33:e14155. doi: 10.1111/dth.14155, PMID: 32770695

[80] Stingeni L Marietti R Bianchi L Guarneri F Ferrucci SM Faraci AG. Patch testing of budesonide in Italy: the SIDAPA baseline series experience, 2018-2019. Contact Dermatitis. (2021) 85:317–23. doi: 10.1111/cod.13873, PMID: 33931866

[81] Brar KK Leung DYM. Eczema complicated by allergic contact dermatitis to topical medications and excipients. Ann Allergy Asthma Immunol. (2018) 120:599–602. doi: 10.1016/j.anai.2018.04.017 29702202

[82] Jacob SE Scheman A McGowan MA. Propylene glycol. Dermatitis. (2018) 29:3–5. doi: 10.1097/DER.0000000000000315 29059092

[83] Coloe J Zirwas MJ. Allergens in corticosteroid vehicles. Dermatitis. (2008) 19:38–42. doi: 10.2310/6620.2008.07054 18346395

[84] González-Muñoz P Conde-Salazar L Vañó-Galván S. Allergic contact dermatitis caused by cosmetic products. Actas Dermosifiliogr. (2014) 105:822–32. doi: 10.1016/j.ad.2013.12.018 24656778

[85] Goossens RA. Allergic contact dermatitis from the vehicle components of topical pharmaceutical products. Immunol Allergy Clin N Am. (2014) 34:663–70. doi: 10.1016/j.iac.2014.04.010, PMID: 25017683

[86] Foti C Romita P Cristaudo A Corazza M Gallo R Massari F. Contact allergy to 3-dimethylaminopropylamine in 5140 consecutive Italian patients: a one-year retrospective multicenter SIDAPA study. Contact Dermatitis. (2020) 82:240–1. doi: 10.1111/cod.13445, PMID: 31785001

[87] Santiago F Andrade P Gonçalo M Mascarenhas R Figueiredo A. Allergic contact dermatitis to shoes induced by dimethylfumarate: a new allergen imported from China. Dermatol Online J. (2010) 16:3. doi: 10.5070/D31N56G6CF 20233560

[88] Stingeni L Neve D Tondi V Bacci M Lisi P. Immunological contact urticaria caused by dimethyl fumarate. Contact Dermatitis. (2014) 71:180–3. doi: 10.1111/cod.12230, PMID: 25155075

[89] Stingeni L Cerulli E Spalletti A Mazzoli A Rigano L Bianchi L. The role of acrylic acid impurity as a sensitizing component in electrocardiogram electrodes. Contact Dermatitis. (2015) 73:44–8. doi: 10.1111/cod.12357, PMID: 25645530

[90] Foti C Lopalco A Stingeni L Hansel K Lopedota A Denora N. Contact allergy to electrocardiogram electrodes caused by acrylic acid without sensitivity to methacrylates and ethyl cyanoacrylate. Contact Dermatitis. (2018) 79:118–21. doi: 10.1111/cod.13015, PMID: 29749023

[91] Zirwas MJ. Contact dermatitis to cosmetics. Clin Rev Allergy Immunol. (2019) 56:119–28. doi: 10.1007/s12016-018-8717-9 30421329

[92] Lisi P Stingeni L Cristaudo A Foti C Pigatto P Gola M. Clinical and epidemiological features of textile contact dermatitis: an Italian multicenter study. Contact Dermatitis. (2014) 70:344–50. doi: 10.1111/cod.12179, PMID: 24392992

[93] Isaksson M Ryberg K Goossens A Bruze M. Recommendation to include a textile dye mix in the European baseline series. Contact Dermatitis. (2015) 73:15–20. doi: 10.1111/cod.12400 25925831

[94] Stingeni L Bianchi L Marietti R Ferrucci SM Zucca M Foti C. Patch testing with textile dye mix in Italy: a 2-year multicenter SIDAPA study. Contact Dermatitis. (2021) 84:265–8. doi: 10.1111/cod.13721, PMID: 33063872

[95] Stingeni L Bianchi L Hansel K Corazza M Gallo R Guarneri F. Italian guidelines in patch testing – adapted from the European Society of Contact Dermatitis (ESCD). G Ital Dermatol Venereol. (2019) 154:227–53. doi: 10.23736/S0392-0488.19.06301-6, PMID: 30717577

[96] Bruynzeel DP Andersen KE Camarasa JG Lachapelle JM Menné T White IR. The European standard series. European environmental and contact dermatitis research group (EECDRG). Contact Dermatitis. (1995) 33:145–8. doi: 10.1111/j.1600-0536.1995.tb00534.x 8565452

[97] Hannuksela M Salo H. The repeated open application test (ROAT). Contact Dermatitis. (1986) 14:221–7. doi: 10.1111/j.1600-0536.1986.tb01229.x 2941220

[98] HER Inc./Mayo Clinic Available at: www.SkinSafeProducts.com

[99] Available at: www.ContactDerm.org

[100] Raff J Elaine Allen I Botto N. Validating responsiveness of a quality-of-life instrument for allergic contact dermatitis. Dermatitis. (2020) 31:209–14. doi: 10.1097/DER.0000000000000542 32049717

[101] Scalone L Cortesi PA Mantovani LG Belisari A Ayala F Fortina AB. Clinical epidemiology of hand eczema in patients accessing dermatological reference centres: results from Italy. Br J Dermatol. (2015) 172:187–95. doi: 10.1111/bjd.13220, PMID: 24974982

[102] Di Agosta E Salvati L Corazza M Baiardini I Ambrogio F Angileri L. Quality of life in patients with allergic and immunologic skin diseases: in the eye of the beholder. Clin Mol Allergy. (2021) 19:26–6. doi: 10.1186/s12948-021-00165-6, PMID: 34930291 PMC8690422

[103] Parikh D Dhar S Srinivas S Rammoorthy R Sarkar R Inamadar A. Treatment guidelines for atopic dermatitis by Indian Society for Pediatric Dermatology task force 2016 - Part-2: topical therapies in atopic dermatitis. Indian *J Paediatr Dermatol*. (2017) 18:274–80. doi: 10.4103/ijpd.IJPD_99_17

[104] Chipalkatti N Lee N Zancanaro P Dumont N Kachuk C Rosmarin D. A retrospective review of dupilumab for atopic dermatitis patients with allergic contact dermatitis. J Am Acad Dermatol. (2019) 80:1166–7. doi: 10.1016/j.jaad.2018.12.048, PMID: 30630024

[105] Muñoz-Bellido FJ Moreno E Dávila I. Dupilumab: a review of present indications and off-label uses. J Investig Allergol Clin Immunol. (2022) 32:97–115. doi: 10.18176/jiaci.0682, PMID: 33661102

[106] Ferrucci S Angileri L Tavecchio S Fumagalli S Iurlo A Cattaneo D. Elevation of periphereal blood eosinophils during dupilumab treatment for atopic dermatitis is associated with baseline comorbidities and development of facial redness dermatitis and ocular surface disease. J Dermatolog Treat. (2022) 33:2587–92. doi: 10.1080/09546634.2022.2049588 35261333

[107] Bangsgaard N Zachariae C Menné T Skov L. Lack of effect of ustekinumab in treatment of allergic contact dermatitis. Contact Dermatitis. (2011) 65:227–30. doi: 10.1111/j.1600-0536.2011.01907.x, PMID: 21718332

